# Possible Nosocomial Transmission of *Pneumocystis jirovecii*

**DOI:** 10.3201/eid1805.111432

**Published:** 2012-05

**Authors:** Céline Damiani, Firas Choukri, Solène Le Gal, Jean Menotti, Claudine Sarfati, Gilles Nevez, Francis Derouin, Anne Totet

**Affiliations:** Centre Hospitalier Universitaire d’Amiens, Amiens, France (C. Damiani, A. Totet);; Université de Picardie-Jules Verne, Amiens (C. Damiani, A. Totet);; Hôpital Saint Louis, Paris, France (F. Choukri, J. Menotti, C. Sarfati, F. Derouin);; Université Paris Diderot, Paris (F. Choukri, J. Menotti, C. Sarfati, F. Derouin);; Centre Hospitalier Universitaire de Brest, Brest, France (S. Le Gal, G. Nevez);; Université de Brest, Brest (S. Le Gal, G. Nevez)

**Keywords:** Pneumocystis jirovecii, genotype, air, transmission, nosocomial, infection, fungi

**To the Editor:** Diversity of genotypes among *Pneumocystis jirovecii* (human-specific *Pneumocystis* species) isolates mainly involves internal transcribed spacer (ITS) loci (*1*). Type Eg, one of the most frequently detected ITS genotypes, has been found worldwide (*2*). The locus of dihydropteroate synthase (DHPS) is also of interest because DHPS is the target of sulfonamides, the main drugs used to treat *Pneumocystis* pneumonia (PCP). Studies of the DHPS locus have found mutations at positions 165 and 171, which confer potentially lower sensitivity to sulfonamides to mutant *P. jirovecii* organisms (*3*).

Airborne transmission of *Pneumocystis* ssp. has been demonstrated among animals and probably occurs among humans (*4*). Reports of clusters of PCP cases in hospitals (*4**,**5*) provide a rationale for considering the possibility of nosocomial *P. jirovecii* infections. Moreover, we recently quantified *P. jirovecii* in the air surrounding patients with PCP (*6*). Our findings suggested that the fungus is exhaled from infected patients and then spreads into their surrounding air.

Because matches of *P. jirovecii* genotypes between pulmonary and air samples would strengthen these findings, we conducted DHPS and ITS typing of *P. jirovecii* isolates from PCP patients and from the air in their close environment. We assayed *P. jirovecii* DNA that we had previously detected in pulmonary samples (bronchoalveolar lavage and induced sputum) from 15 PCP patients and in 15 air samples collected 1 meter from each patient’s head (*6*).

ITS genotyping was based on sequence analysis of ITS 1 and 2 regions after amplification with a nested PCR, cloning, and sequencing, as described (*7*). ITS alleles were identified by using the typing system by Lee et al. (*2*). DHPS genotyping was based on a PCR restriction fragment-length polymorphism assay that enables detection of mutations at positions 165 and 171, as described (*8*).

Among the 15 pulmonary samples, ITS genotyping was successful for all 15; among these, 8 ITS genotypes were identified (Table). Type Eg was most frequently identified. Mixed infections, which correspond to detection of >1 genotype in a given sample, were detected in 5 samples. DHPS genotyping was successful for all 15 pulmonary samples. A wild genotype was identified in 9 samples, a 165 mutant genotype in 1 sample, and a 171 mutant genotype in 2 samples. Mixed infections were identified in the 3 remaining samples.

**Table:** Genotyping of *Pneumocystis jirovecii* in pairs of pulmonary and air samples from 15 patients with *Pneumocystis* pneumonia*

Patient no.†	No. days between pulmonary and air sampling	ITS genotype (no. sequenced clones)‡			DHPS genotype§	
		Pulmonary sample	Air sample		Pulmonary sample	Air sample
1	6	Gg, Fg (3)¶	ND		Wild	ND
2	1	Ih (3)	Eg (3)		Wild	ND
4	0	Gg (3)	Gg (3)		Wild	Wild
5	0	Eg (3)	Ec, Eg (3)¶		Wild	Wild
6	0	Eg (3)	Eg (3)		Mutant 171#	Mutant 171
7	1	Eg (3)	ND		Wild	Wild
8	0	Eg (2)	Eg (1)		Wild	ND
10	0	Eg (3)	ND		Mutant 171	ND
11	0	Be, Ec (3¶)	Ec (3)		Mutant 165**	Mutant 165
13	2	Eg (3)	ND		Wild	ND
15	0	Eg, Fg (3)¶	ND		Wild + mutant 171	ND
16	0	Eg (3)	ND		Wild	Wild
17	1	Ie, Ih (2)	ND		Wild	ND
18	1	Bl (3)	Bl (3)		Wild + mutant 165	ND
19	0	Eg, Bl (3¶)	ND		Wild + mutant 165	ND

*ITS, internal transcribed spacers; DHPS, dihydropteroate synthase; ND, not determined. †Patients are numbered as described in (*6*). Pulmonary samples were bronchoalveloar lavage specimens for patients 2, 4, 6, 7, 8, 10, 11, 18, and 19 and induced sputum specimens for patients 1, 5, 13, 15, 16, and 17. ‡*P. jirovecii* ITS genotype identification using sequence analysis with a prior cloning step and applying the score by Lee et al. (*2*). §*P. jirovecii* DHPS genotype identification using a PCR restriction fragment length polymorphism assay (*8*). ¶Major ITS genotype, as identified in 2 of 3 clones. #Mutant genotype with the mutation at position 171. **Mutant genotype with the mutation at position 165.

Among the 15 room air samples, ITS genotyping was successful for 7; among these, 4 ITS genotypes were identified (Table). Type Eg was again most frequently identified. A mixed infection was detected in 1 of the 7 samples. These results enabled us to compare ITS genotypes for 7 pairs of pulmonary and air samples. A full match was found for 4 (57.1%) pairs of samples, and a partial match, defined as at least 1 common genotype for pulmonary and air samples in mixed infections, was found for 2 (28.6%) pairs. No matches were found for the remaining pair of samples. DHPS genotyping was successful for 6 of the 15 air samples. A wild genotype was identified in 4 samples, a 165 mutant genotype was identified in 1 sample, and a 171 mutant genotype was identified in 1 sample. These results enabled us to compare DHPS genotypes for 6 pairs of samples. A full match was found for these 6 pairs. DHPS and ITS genotype matches were found for 4 pairs.

Several lines of evidence suggest that *P. jirovecii* is exhaled by infected patients and transmitted by the airborne route to susceptible persons (*4*). In the study reported here, ITS or DHPS genotype matches between pairs of pulmonary and air samples are consistent with the possibility that *P. jirovecii* organisms in the air originated from patients. DHPS mutants were detected in 6 (40%) of the 15 pulmonary samples; none of the15 patients had received sulfonamide treatment at the time of PCP diagnosis. These results were not unexpected because frequency of finding DHPS mutants in PCP patients in Paris who had no prior sulfonamide treatment is high (*8*). The exhalation of DHPS mutants from infected patients can spread potentially sulfonamide-resistant organisms.

Matches of *P. jirovecii* genotypes in pairs of pulmonary and room air samples argue in favor of *P. jirovecii* exhalation by infected patients. The exhalation of *P. jirovecii* organisms emphasizes the risk for their nosocomial transmission. Our data provide additional arguments in favor of the application of measures to prevent the airborne transmission of *P. jirovecii* in hospitals.
